# Change in popliteal angle and hamstrings spasticity during childhood in ambulant children with spastic bilateral cerebral palsy. A register-based cohort study

**DOI:** 10.1186/s12887-019-1891-y

**Published:** 2020-01-08

**Authors:** Merete Aarsland Fosdahl, Reidun Jahnsen, Are Hugo Pripp, Inger Holm

**Affiliations:** 1 Department of Clinical Neuroscience for Children, Division of Pediatric and Adolescent Medicine, Oslo University Hospital, Oslo, Norway; 2 Department of Clinical Neuroscience for Children, The Cerebral Palsy Follow-up Program (CPOP), Division of Pediatric and Adolescent Medicine, Oslo University Hospital, Oslo, Norway; 3 Oslo Centre of Biostatistics and Epidemiology, Research Support Services, Oslo University Hospital, Oslo, Norway; 4 Division of Orthopaedic Surgery, Department of Research and Development, Oslo University Hospital, Oslo, Norway; 5 Medical Faculty, Department of Interdisciplinary Health Sciences, University of Oslo, Oslo, Norway

**Keywords:** Cerebral palsy, Hamstrings, Popliteal angle, Spasticity, Cohort

## Abstract

**Background:**

Muscle contractures are developing during childhood and may cause extensive problems in gait and every day functioning in children with cerebral palsy (CP). The aim of the present study was to evaluate how the popliteal angle (PA) and hamstrings spasticity change during childhood in walking children with spastic bilateral CP.

**Methods:**

The present study was a longitudinal register-based cohort study including 419 children (1–15 years of age) with spastic bilateral CP, gross motor function classification system (GMFCS) level I, II and III included in the Norwegian CP Follow-up Program (CPOP). From 2006 to 2018 a total of 2193 tests were performed. The children were tested by trained physiotherapists yearly or every second year, depending on GMFCS level and age. The PA and the hamstrings spasticity (Modified Ashworth scale (MAS)) were measured at every time point. Both legs were included in the analysis.

**Results:**

There was an increase in PA with age for all three GMFCS levels with significant differences between the levels from 1 up to 8 years of age. At the age of 10 years there was no significant difference between GMFCS level II and III. At the age of 14 years all three GMFCS levels had a mean PA above 40° and there were no significant differences between the groups. The hamstrings spasticity scores for all the three GMFCS levels were at the lower end of the MAS (mean < 1+), however they were significantly different from each other until 8 years of age. The spasticity increased the first four years in all three GMFCS levels, thereafter the level I and II slightly increased, and level III slightly decreased, until the age of 15 years.

**Conclusion:**

The present study showed an increasing PA during childhood. There were significantly different PAs between GMFCS level I, II and III up to 8 years of age. At the age of 14 years all levels showed a PA above 40°. The spasticity increased up to 4 years of age, but all the spasticity scores were at the lower end of the MAS during childhood.

## Background

Muscle shortening and decreased joint motion in the lower extremities are frequently recognised in children with spastic cerebral palsy (CP) [1–3], and gradual deterioration of gait function and performance of everyday activities is common [4, 5]. CP is caused by injury or insult to the immature brain, and the pathology in the brain is permanent and non-progressive [6, 7]. The primary manifestation of the neurological insult to the brain causes loss of selective motor control, muscle imbalance, and muscle tone abnormalities. These impairments frequently result in secondary conditions like muscle contractures, reduced joint motion and balance, often affecting function and everyday life [6, 7]. Many of these secondary complications are developing slowly over years, hence the related functional complications will be gradually recognized [2, 5]. The gradual deterioration seems to worsen by increasing age and lower functional levels as measured by the Gross Motor Function Classification System (GMFCS) [2, 8]. Early detection and identification of secondary complications are important [9] and may give health professionals and parents opportunities to prevent or limit an expected negative development.

Systematic follow-up programs including early detection and treatment of deteriorating joint motion and musculoskeletal functioning are assumed to enable prevention of permanent disability, and postponement or avoidance of surgical procedures [10]. In Sweden a follow-up program (CPUP) for children with CP, including non-surgical treatment modalities for prevention of deteriorating joint motion, showed that the number of surgeries for contractures decreased by 65% over a 10 year period (1994–2004) [9, 10]. A recent prospective cohort study showed that joint contractures may hamper long term gross motor progress, while intensive training programs (≥3 times per week) enhance gross motor progress [11, 12]. Hence avoiding joint contractures and reduced range of motion (ROM) should be important treatment goals and is strongly emphasised in the literature [6, 9, 12, 13]. Due to increased spasticity, muscle stiffness, and contractures in the distal muscles compared to the proximal muscles, the treatment programs for the young ambulant children include modalities to avoid pes equinus and toe walking [6]. It is well documented that the hamstrings muscles become shortened and less flexible during childhood [2, 5, 14]; however, how the hamstrings contractures develop during childhood is not documented to the same extent as contractures of triceps surae. The hamstrings is a group of muscles crossing both the hip and the knee joints, impacting both joints by rotating the pelvis backwords and flexing the knees [6]. In addition to factors like gastrocnemius spasticity, triceps surae weakness, generalised muscle weakness and mal-alignment, short and spastic hamstrings may contribute to flexed knee gait and crouch [15]. Crouch is the most common and severe gait abnormality in ambulant children with CP [6] resulting in abnormal mechanical loads on the knee, hip, and ankle joints, which may cause joint pain, joint degeneration, and bony deformities [16].

One frequently used method for evaluating hamstrings length is measuring the popliteal angle (PA). Even though there are some controversies [17], studies have shown that hamstrings length correlates moderately with knee flexion during stance and at initial heel contact, and a short hamstrings is linked to shorter stride length and a backward rotation of the pelvis [15]. There is also a tenfold increased risk of knee flexion contracture when a short hamstring is present (PA > 60°) [18]. To be able to prevent shortening of the hamstrings, there is a need for knowledge about how the length of the muscle group changes during childhood.

Hamstrings spasticity may also affect the active knee ROM [19]. Previous studies on children with CP and triceps surae have shown that the spasticity is changing during childhood [20, 21]. To our knowledge, there is no longitudinal study which has evaluated these changes in children with CP.

The aim of the present study was to analyse how the PA and hamstrings spasticity change during childhood in walking children with spastic bilateral CP (SBCP), GMFCS level I, II, and III.

## Methods

The present study was a longitudinal register-based cohort study including data from the Norwegian CP Follow-up Program (CPOP) [22]. CPOP is a consent based program, where the children are followed up and tested according to a standardised protocol with fixed intervals throughout the childhood. The main purpose of the program is to identify and contribute to prevent secondary complications at an early stage. The CPOP was launched in 2006 and included children from the South-Eastern health region of Norway. From 2010 children from the rest of the country were also included in the register. In 2017 the program included about 90% of all children diagnosed with CP in Norway [23]. The children are tested from the age of one year or from the time of the CP-diagnosis (mean age 25 months) (20). Data are collected each year until the age of 6, and thereafter yearly at GMFCS level II-V and every second year at GMFCS level I (yearly before 2015). There were 419 eligible children with SBCP, GMFCS I, II and III, aged 1 to 15 years eligible for inclusion in the present study.

**Fig. 1:**
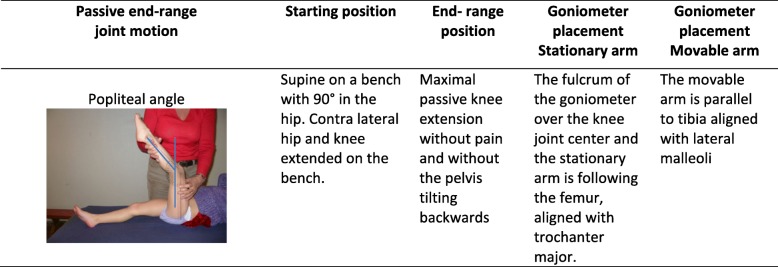
Assessment of the popliteal angle. End-range position measuring the popliteal angle. (With permission from CPOP [24])

Variables included in the present study were age, gender, GMFCS level, PA and hamstrings spasticity. GMFCS [8] is an age-related, five level scale where levels I-III include walkers (level III is dependent on hand-held walking devices) and level IV and V are non-walkers. The GMFCS level is re-evaluated at every assessment. The measurements are performed in regional paediatric rehabilitation units by two persons following the CPOP manual [24, 25]. In the CPOP [22] the changes in hamstrings length are followed by measuring the PA measured by a goniometer (Fig. 1). This method is widely used as a measure of hamstrings length in children with CP, both in clinical and research settings [1, 2, 5, 15, 17]. However, the validity [26] and reliability [27–30] of the PA measurements are discussed. The inter- and intra- observer reliability has been shown to be low [27–30]. To optimise the reliability, the CPOP-follow-up program provides a detailed test protocol and when possible, the assessments are supposed to be performed by the same two trained assessors at each test session [24].

The assessments are categorised into three levels of severity. The first level (≤ 39°) indicates a satisfactory passive PA, the second level (40–49°), indicates a reduced PA and a need of more frequent follow-ups and consideration of treatment initiatives. The third level (≥ 50°) indicates a PA which needs intervention. Both PA and hamstrings spasticity were assessed in a supine position with the hip in 90° of flexion and the contralateral hip extended on the bench (Fig. 1). Hamstrings spasticity was measured according to Modified Ashworth Scale (MAS) [31]. Due to the nature of spasticity, the validity and reliability is moderate, and controversies about the interpretation of the tests exist. Nevertheless, it is the most frequently used scale for clinical evaluation of spasticity in CP [20, 21, 32–34]. The spasticity is graded from 0, normal muscle tone, 1 to 1+, small increase in muscle tone, and 2 to 4, large increase in muscle tone [31]. The intention of the modified version of MAS, was to improve the sensitivity in the lower end of the scale [31].

The completed paper protocols were posted by mail to the CPOP secretariat, where the data were plotted into an electronic database (Medinsight) [35]. Data in the present study were exported from Medinsight to Stata 15.0 (StataCorp LLC, College Station, TX). The statistical calculations were based on measurements from both legs.

### Statistical analysis

Data were described with number of observations and percentage or mean and standard deviation as appropriate. We used a multivariable fractional polynomial linear regression model to fit age curves for the GMFCS levels. The model contained age (as continuous variable), GMFCS level (as categorical/dummy variable) and the interaction between age and GMFCS level as independent variables. In the model estimation, we used a robust standard error (clustered sandwich estimator) on participant due to the repeated measurement data. Both legs were included in the analysis, and the model has taken into account the dependency between legs in the same individual. For model selection, we used the mfp command in Stata which selects a multivariable fractional polynomial model in linear regression analysis (regress command in Stata) that best predicts the outcome variable using the default closed-test procedure algorithm [36]. The analyses was using the total cohort, including children treated with Botulinum neurotoxin-A (BoNT-A) and oral baclofen. Two additional analyses were performed; with children treated with hamstrings tenotomy and children treated with intrathecal baclofen pump (ITB)/selective dorsal rhizotomy (SDR) respectively excluded.

The linear prediction with 95% confidence interval for GMFCS level I, II and III was graphically presented. All statistical analysis was conducted with Stata 15.0.

## Results

**Fig. 2:**
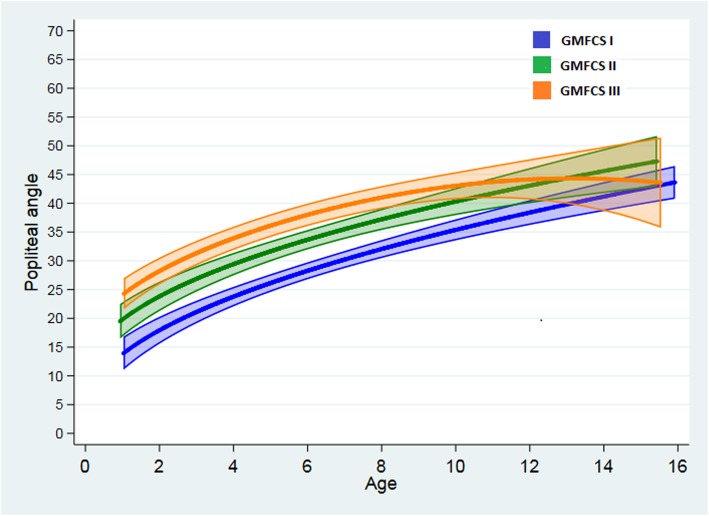
Change in popliteal angle. Change in popliteal angle at GMFCS level I, II and III, from 1 to 16 years of age

Data were obtained from 419 children with BSCP, GMFCS I, II and III; 161 (38%) girls and 258 (62%) boys (Table 1). In total there were 2193 assessments, from one to 16 assessments per child, mean 4.3 (± 2.9), and 3.8 (± 2.7), 5.1 (±3.4), 4.1 (± 2.7) at GMFCS level I, II, III, respectively. Both legs (4386 measures) were included in the curve estimates. The distribution according to GMFCS levels and gender is presented in Table 1. Figure 2 and Table 2 show a parallel increase in PA with age for the three GMFCS levels with significant difference between all the GMFCS levels from 2 to 8 years of age (*p* ≤ 0.005) (Fig. 2 and Table 2). At GMFCS level I and II PA were increasing by a mean of 4–5° every second year throughout the age span. In contrast, PA at GMFCS level III was levelling off with only a minimal increase after 10 years. At 10 years there was no significant difference in PA between GMFCS level II and III, and at 14 years there was no significant difference in PA between any of the three GMFCS levels (Fig. 2 and Table 2).

**Table 1:** Demographic data

	N (%)	N (%) girls	N (%) boys
GMFCS I	198 (47%)	75 (38%)	123 (62%)
GMFCS II	116 (28%)	44 (38%)	72 (62%)
GMFCS III	105 (25%)	42 (40%)	63 (60%)
Total	419	161 (38%)	258 (62%)

GMFCS: Gross Motor Function Classification System

**Table 2:** Distribution of GMFCS-levels in relation to popliteal angle measurements during childhood

	2 years	4 years	6 years	8 years	10 years	12 years	14 years
GMFCS I	17.9 (15.6–20.3)	23.8 (22.1–25.5)	28.3 (26.8–29.7)	32.1 (30.5–33.6)	35.4 (33.6–37.2)	39.4 (36.2–40.6)	41.2 (38.6–43.7)
GMFCS II	23.8 (21.3–26.4)	29.4 (27.5–31.4)	33.7 (32.0–35.4)	37.2 (35.4–39.1)	40.3 (37.9–42.7)	43.1 (40.0–46.1)	45.6 (41.8–49.6)
GMFCS III	28.33 (25.9–30.55)	33.9 (31.9–36.0)	38.0 (36.1–40.0)	41.0 (39.0–43.0)	43.1 (40.7–45.4)	44.2 (40.6–47.7)	44.3 (38.7–49.9)

Mean popliteal angle (95% CI) over time divided by the gross motor function classification system (GMFCS) levels I, II and III

GMFCS: gross motor function classification system; CI: confidence interval

**Fig. 3:**
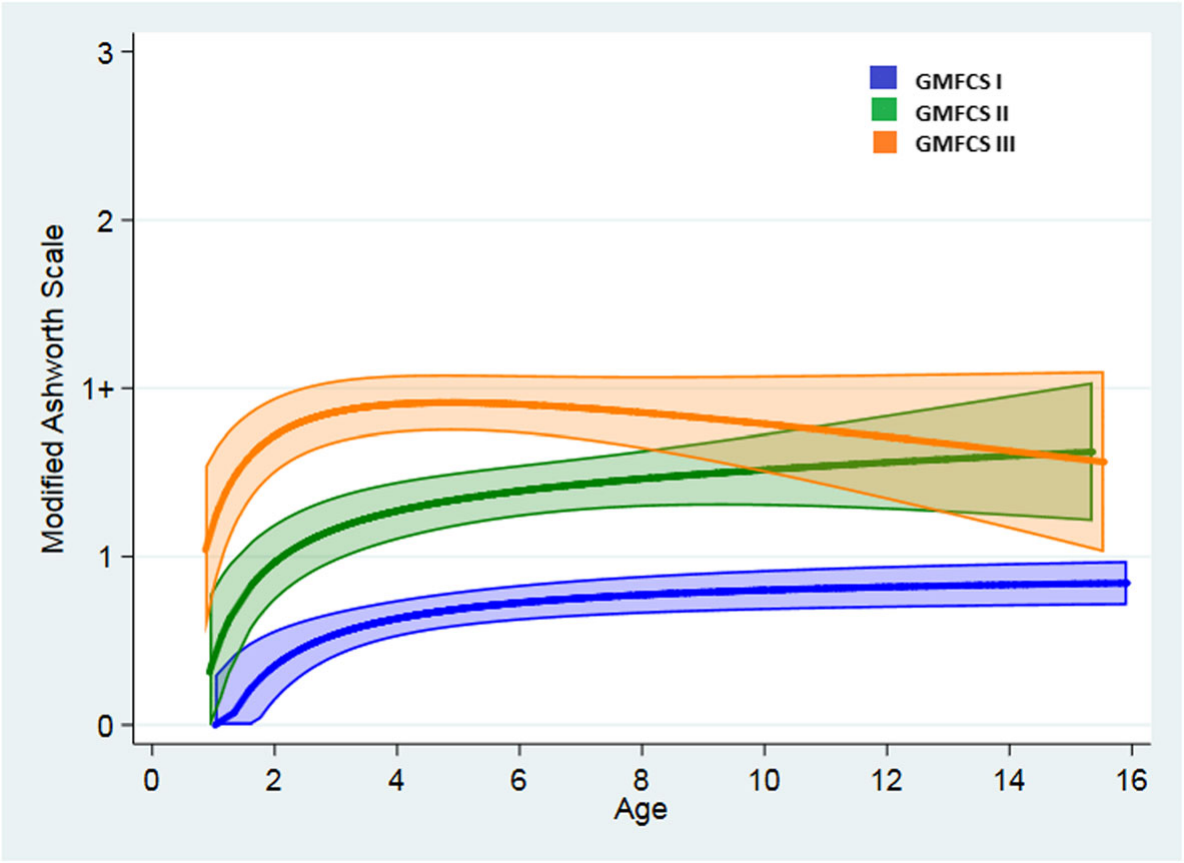
Change in the modified Ashworth Scale. Change in modified Ashworth Scale at GMFCS level I, II and III, from 1 to 16 years of age

The hamstrings spasticity curve estimates for all the three GMFCS levels were low (0–1+) measured by the MAS (Fig. 3). However, all three levels showed steep parallel curves during the first four years, with significant different MAS scores (*p* < 0.005) between the levels. At the age of 4 years the curves at GMFCS level I and II were levelling off, followed by minimally increasing curves until the age of 15 years. At GMFCS level III the spasticity curve peaked between 4 and 6 years of age before slightly decreasing (Fig. 3). There were significant differences between the MAS curves for all the GMFCS levels (*p* < 0.005) up to 8 years, but at the age of 10 years the MAS curves at GMFCS level II and III were overlapping and no longer significantly different (Fig. 3).

Additional analyses were performed by excluding those children who had undergone hamstrings tenotomy (*n* = 29), or ITB/SDR (*n* = 14). The mean age at surgery was 8.6 years (±2.5) and 5.3 years (±1.7) respectively. The analysis excluding those who had undergone hamstrings tenotomy had no influence on the curve patterns at GMFCS level I and II. At GMFCS level III, however, the analysis showed a decrease in the PA curve from the age of 9 years. For the MAS at GMFCS level III, the results showed a steeper curve from the age of 6 years, ending below MAS 1 at the age of 14 years. Excluding those children who had received SDR/ITB did not influence either the PA or the MAS curves.

Fifty-eight percent of the children in the cohort received one or more medical or surgical interventions on the lower extremities throughout the observation period (Table 3). At the GMFCS levels I, II and III orthopaedic surgery were performed in 12, 30, and 42%, and BoNT-A injections were given in 44, 66, and 63%, respectively. In addition, 87% of all the children received physiotherapy monthly or more frequently, 66% received physiotherapy one or more times per week, and 72% used some kind of orthoses, mostly ankle-foot-orthoses.

**Table 3:** Type and number of interventions performed at each GMFCS-level during childhood

	n	Total number of surgeries In lower extremities. n (%)	Hamstrings tenotomy n (%)	BTX lower extr. n (%)	BTX Hamstring n (%)	SDR/ITB n (%)	BTX/Surgery/ITB/SDR n (%)
GMFCS I	198	22 (11.8%)	5 (2.7%)	81 (43.6%)	24 (12.9%)	0/0	86 (43.4%)
GMFCS II	116	35 (30.2%)	7 (6.0%)	77 (66.4%)	25 (21.6%)	3/0 (2.6%/0%)	81 (69.8%)
GMFCS III	105	44 (41.9%)	17 (16.2%)	66 (62.9%)	37 (35.2%)	9/2 (8.6%/1.9%)	76 (72.4%)
Total	419	101 (24.1%)	29 (6.9%)	224 (53.5%)	86 (20.5%)	12/2 (2.9%/0.5%)	243 (58%)

GMFCS: gross motor function classification system, Extr.: extremities, SDR: selective dorsal rhizotomy, ITB: intrathecal baclofen. The (%) is calculated from the number children at each GMFCS level

An intervention is only registered once pr child, however, one child may have had more than one type of intervention/surgery

## Discussion

To our knowledge the current study is the first study to document how the PA and the hamstrings spasticity changes during childhood in a cohort of children with SBCP, GMFCS levels I, II, and III. The estimates from the statistical model showed that the PA was increasing during childhood, and that the GMFCS levels evolved with somewhat different patterns (Fig. 2). However, at 14 years of age no significant differences between the GMFCS levels were found. The hamstrings spasticity curves also changed during childhood, and as for the PA the patterns were different at the three GMFCS levels (Fig. 3). The spasticity curves increased throughout childhood, but were categorised as “a small increase in muscle tone” (mean < 1+) at all three GMFCS levels. At 10 years of age there was no significant difference in MAS scores between GMFCS levels II and III (Fig. 3).

For the PA curves there were 5° estimated differences between the GMFCS levels, and the significant differences seemed stable until the age of 8 years (Fig. 2 and Table 2). These findings are partly in line with previous published studies [1, 2]. At GMFCS level III the mean PA reached 41° (95% CI 39–43) at 8 years of age, which according to CPOP [22] indicates an increased PA, a need for more frequent follow-ups, and consideration of intervention initiatives. However, from the age of 12 years, the curve at GMFCS level III was levelling off. The curves at GMFCS levels I and II showed a slightly different slope, reaching 40° at 14 and 10 years respectively, and continuing to increase. The upper part of the CI band of GMFCS levels II and III were reaching 50° at about 14 years of age, which according to CPOP and others [6] indicates a need for treatment initiatives [22]. At GMFCS level III, the CI band was wide in the highest age groups, overlapping the CI of both GMFCS levels I and II, indicating a statistical uncertainty, and no significant difference between the groups. This may be explained as a statistical artefact due to fewer children in the oldest age groups, especially at GMFCS level III. One explanation why the PA at GMFCS level III was levelling off may be seen in the hamstrings spasticity curves (Fig. 3), which were peaking at the age of 6 and then descending. This decreased spasticity after the age of 6 years may indicate a decreased risk of contracture [33]. Seventy-two percent of the children at GMFCS level III had received BoNT-A, ITB, SDR, or surgery in the lower extremities during childhood (Table 3). In addition there were more interventions targeting the hamstrings at GMFCS level III compared to GMFCS levels I and II (Table 3), which may also have affected the shape of the curve. The separate analysis, excluding children who had undergone hamstrings tenotomy showed a decrease in both the MAS and PA curves at 6 and 9 years of age respectively, but only at GMFCS level III. No changes at GMFCS level I and II was probably due to few children who had undergone hamstrings tenotomy at these levels (Table 3). The hamstrings tenotomies were performed at a mean age of 8.6 years (±2.5). At the age of 9 years, the PA curve representing GMFCS level III, (Fig. 2) stopped to increase at about 43° and then levelled off, which may indicate that it was the children with the biggest PA who had received hamstrings surgery. Nordmark et al. [2], who studied all CP subgroups (spastic uni-, and bilateral CP, ataxia, and dyskinesia) of children with CP in Sweden also described a mean increase in the PA from 1 to14 years at all GMFCS levels. The curve estimates at GMFCS levels II and III were almost identical to our findings from about 5 to 10 years of age, however Nordmark did not describe a levelling or decrease of PA in the oldest age span at GMFCS level III as shown in the present study. They also performed additional analyses excluding the children (*n* = 6) who had undergone a hamstrings tenotomy, and the exclusion did not influence the PA curve. Reasons for the different findings in the two studies may be that they included all subgroups of CP and that fewer children had undergone a hamstrings tenotomy [2].

McDowell et al. [1] studied a sample of 178 children (4–17 years of age) with spastic CP and reported an increase in PA with increasing age and GMFCS level, with a higher PA in those having a bilateral involvement compared to those who were unilaterally affected. Compared to the findings in the present study, they found higher PAs at all GMFCS levels. One explanation might be that they excluded children who had undergone surgery the last year and those who had BoNT-A treatment the latest 6 months.

From 1 to 10 years of age the PA curves, GMFCS level I, showed CIs significantly narrower than GMFCS levels II and III (Fig. 2). One explanation might be that the level I group was the biggest group including 47% of the children, which might to a certain degree influence the distribution of the results. There was a continuous increase in the PA throughout childhood, reaching 41° at 14 years of age (Table 2, Fig. 2). This indicates that the PA in GMFCS level I increased despite of relatively good function, and a low spasticity level (Fig. 3). In comparison to studies reporting the PA in typically developing (TD) children, the children at GMFCS level I had a 10° to 15° higher PA. Mc Dowel et al. [1] reported a PA of 26° (±11) in TD children 4–10 years of age (*n* = 39) and 32° (±10) in TD children 11–17 years of age (*n* = 29). Moon et al. [37] reported in a group of TD adolescents 13–20 years old (*n* = 26) a PA of 34° (±10). Both studies showed increasing PAs with increasing ages, however, in the present study the change with age at GMFCS level I was more pronounced. As for the TD children, age seemed to be an important factor for the evolvement of the PA in walking children with CP. Rose et al. [5] followed 18 children with bilateral CP, mainly GMFCS levels I and II (mean age at inclusion 7.7 years) to evaluate the effect of time on their gait. The children performed 3D-gait analysis twice, the time intervals differed from 4.3–9.3 years. The results did not show any significant change in PA and flexed knee gait until the observation period reached at least 6 years. Rethlefsen et al. [38] studied 1005 gait records retrieved from ambulant children with CP. They reported that the odds for having excessive knee flexion in stands increased with increasing age at GMFCS level I, II and III, but only reaching significance at GMFCS level I.

In the present study the curve estimates for the hamstrings spasticity development the first 4 years were steep for all GMFCS levels (Fig. 3), which indicates a rapid change in spasticity during the first years of life, which is in line with previous published findings [6, 21]. However, the spasticity was significantly different in the three GMFCS levels. Compared to the PA curves (Fig. 2) the spasticity curves (Fig. 3) were steeper in the youngest age groups. The peak point of the spasticity curve at GMFCS level III was at the age of about 6 years, followed by a slightly decreasing curve. At the GMFCS levels I and II the curves continued to increase throughout the childhood, however the mean MAS was at the lower end of the spasticity scale (< 1+) at all GMFCS levels. Lindèn et al. [20] performed a register-based prospective cohort study including 4162 children with CP, between 0 and 15 years of age. The analyses included children treated with BoNT-A and oral baclofen and they also performed separate analyses for each GMFCS level. Additional analyses excluding ITB, SDR and Achilles tendon lengthening were performed and no change was found. They reported increased spasticity in the gastrocnemius-soleus muscle up to the age of 5 and thereafter a decreasing muscle tone up to 15 years of age in all CP subtypes. These findings correspond partly with our findings. Linden et al. [20] showed that the spasticity increased until the age of about 5 years for GMFSC level I, II and III and decreased until the age of 15 years. In the present study we found the same increasing tendency in hamstrings spasticity up to 5–6 years of age for GMFCS I, II and III. The spasticity pattern at GMFCS level III from the present study and Linden’s study showed almost the same pattern; the spasticity are decreasing from the age of about 5–6 years until 15 years of age. However, in the present study the spasticity at GMFCS levels I and II (Fig. 3), increased up to the age of 15 years, most pronounced at GMFCS level II. This indicates that the hamstrings spasticity at these two GMFCS levels seems to show a different longitudinal pattern compared to the pattern reported for the gastrocnemius-soleus muscle group [20].

Muscle contractures in CP has generally been associated with the presence of spasticity [7]. Later research draws a more complex picture, also involving impairment of muscle growth and altered muscle adaptation [39]. Hägglund and Wagner [33] found a relationship between spasticity in the gastrocnemius-soleus muscles and the development of contractures in the gastrocnemius-soleus muscle. In the present study, we also found that the PA curves (Fig. 2) and the hamstrings spasticity curves (Fig. 3) had quite identical shapes, especially at GMFCS levels II and III. At the GMFCS level I, the increasing spasticity after the age of 4 was modest (MAS < 1) (Fig. 3), however, the PA curve (Fig. 2) at the GMFCS level I had the highest increment of the three levels presented in this study (Fig. 3). This may indicate that there are additional factors than an increased stretch reflex registered as spasticity contributing to muscle contractures [14, 26, 40–42]. Reduced active terminal knee extension, either due to reduced selective motor control, muscle weakness or immobilization, may be contributing factors [6]. In addition Gough and Shortland [39] suggested that in CP there might be multifactorial impairments of muscle growth which may lead to impaired muscle adaptation during growth [39]. Recent published papers have also shown increased arrangement of collagen in the extra cellular matrix, and factors within the contractile elements in the muscles which may contribute to muscle contractures [14, 26, 40, 42].

In the present cohort a high rate of medical, surgical and physiotherapy interventions had been implemented (Table 3). The high frequency of procedures makes it difficult to assess the natural course of the PA. The reason for the changes is probably due to both natural growth and maturation and an effect of the interventions received during childhood, and in general, it is the most affected children who receive BoNT-A, ITB, SDR and orthopaedic surgery.

Previous studies evaluating reliability of goniometric joint measurements in children with CP [27, 28] have reported big measurement errors when measuring PA. To minimize the influence of confounding factors, and narrow the variability in the measurements, a written standardised protocol as well as trained assessors and, when possible, the same assessor over time has been underlined as important in CP [32]. In the yearly routine for collecting data, written information was distributed from the CPOP [24], and the assessors were trained and experienced physiotherapists. However, to have the same assessor for each child over several years was not always possible. The big number of tests in the current study should limit the variability, but must be taken into account in the interpretation of the results.

The complexity of muscle spasticity makes it difficult to quantify. Several tests exist, however none of them seem to be superior, and validity and reliability are discussed [32]. However, in clinical studies of neurological diseases and injuries the MAS [31] is the most frequently used instrument for assessing spasticity [21, 32]. The MAS added one score level (1+) at the lower end of the original Ashworth scale because the lower end of the spasticity score is more frequently seen in less involved children [18, 31]. This is in line with the results in the present study (Fig. 3).

There were some limitations to this study. The children were tested with different time intervals according to age and GMFCS levels. They were tested each year until the age of 6 years, and thereafter yearly at GMFCS levels II-III and every second year at GMFCS level I. This may indicate uncertainty in the oldest ages at GMFCS I. However, GMFCS level I is the biggest group, including 47% of the children in this cohort. There were fewer children in the oldest and youngest age groups, especially at GMFCS level III, making the confidence intervals wider and the estimates less reliable at this level (Figs. 2 and 3).

## Conclusion

The present register-based cohort study, including children with SBCP, demonstrated that there was an increasing PA by age at GMFCS levels I, II and III. The PA between the GMFCS levels was significantly different during the early childhood, however, at the age of 14 years no significant difference was found. The hamstrings spasticity increased rapidly the first 4 years for children at all GMFCS levels, but the spasticity level was significantly different between the groups. After peaking at about 6 years, the spasticity at GMFCS level III was decreasing, but at GMFCS level I and II the spasticity continued to increase, however the mean MAS never pass category 1+ (small increase in muscle tone) for any of the GMFCS levels.

The results from the present study may have implications for clinical decision-making. The findings that the PA and the hamstrings spasticity seemed to increase during adolescence indicate that awareness on maintaining hamstrings length already at an early age, also for the less involved children, may be important.

## Data Availability

The data that support the findings of this study are available from the Cerebral Palsy Follow –up Program (CPOP), but restrictions apply to the availability of these data, which were used under license for the current study, and so are not publicly available. Data are however available from the authors upon request and with permission of CPOP.

## Abbreviations

BoNT-A: botulinum neurotoxin-A
CP: cerebral palsy
CPOP: Norwegian CP follow-up program
CPUP: Swedish CP follow-up program
GMFCS: gross motor function classification system
ITB: intrathecal baclofen
MAS: modified Ashworth scale
PA: popliteal angle
ROM: range of motion
SBCP: spastic bilateral CP
SDR: selective dorsal rhizotomy
TD: typically developed
